# Case Report: Synergistic multimodal therapy in SMARCA4-deficient undifferentiated tumor: integrating chemotherapy, anti-angiogenesis immunotherapy, and radiotherapy for enhanced outcomes

**DOI:** 10.3389/fonc.2025.1599569

**Published:** 2025-09-05

**Authors:** Fang Xie, Yuming Jia, Kaijian Lei, Zhongming Wang, Wei Zhang, Shiyu Zheng, Daohong Kan

**Affiliations:** ^1^ Department of Oncology, The Second People’s Hospital of Yibin, Yibin, Sichuan, China; ^2^ Department of Internal Medicine, Gongxian Hospital of Traditional Chinese Medicine, Yibin, Sichuan, China; ^3^ Department of Rehabilitation, Gongxian Hospital of Traditional Chinese Medicine, Yibin, Sichuan, China; ^4^ Department of Nuclear Medicine, The Second People’s Hospital of Yibin, Yibin, Sichuan, China; ^5^ Department of Burn and Plastic Surgery, The Second People’s Hospital of Yibin, Yibin, Sichuan, China

**Keywords:** SMARCA4-deficient undifferentiated tumor, 18 F-FDG PET/CT, multimodal therapy, anti-angiogenic therapy, PD-L1 inhibitor, chemotherapy

## Abstract

SMARCA4-deficient undifferentiated tumor is a rare and aggressive malignancy with a poor prognosis, often challenging to diagnose and stage due to its non-specific clinical and imaging features. Herein, we present a case of a 74-year-old male patient initially evaluated for a traumatic knee injury, which serendipitously led to the discovery of a thoracic malignancy. Two FDG PET/CT scans played pivotal roles in initial staging and post-treatment response assessment, guiding multimodal therapy combining chemotherapy, immunotherapy, anti-angiogenesis, and radiotherapy. Sequential PET/CT imaging demonstrated metabolic regression of the primary tumor and metastatic lesions following chemotherapy combined with anti-angiogenic therapy (anlotinib) and PD-L1 inhibition (benmelstobart), followed by consolidative radiotherapy. The treatment achieved complete remission (CR) with sustained disease control at 8 months. This case highlights the potential of a mechanistic-driven, multimodal strategy to overcome therapeutic resistance in SMARCA4-deficient undifferentiated tumor. It underscores the need for further exploration of synergistic regimens in this molecular subset.

## Introduction

SMARCA4-deficient undifferentiated tumor is a rare and clinically aggressive malignancies characterized by the inactivation of the SMARCA4 gene (BRG1), a key regulator of chromatin remodeling within the mammalian switch/sucrose non-fermentable (SWI/SNF) complex (1, 2). Histologically, these tumors display undifferentiated or rhabdoid morphology, often resembling poorly differentiated carcinomas, sarcomatoid mesotheliomas, or other high-grade sarcomas (3, 4). This histologic ambiguity, combined with frequent tumor necrosis, presents significant diagnostic challenges. Conventional imaging modalities, such as contrast-enhanced CT, often cannot distinguish viable tumor tissue from necrotic zones, complicating biopsy site selection and delaying pathologic confirmation. These tumors predominantly affect middle-aged to elderly males with a history of heavy smoking and frequent underlying pulmonary emphysema (5).

The diagnostic dilemma is exemplified in the present case. Initial CT-guided biopsy targeting a large anterior mediastinal mass yielded necrotic tissue devoid of diagnostic material—a scenario frequently encountered in SMARCA4-deficient undifferentiated tumors due to their propensity for central necrosis (5, 6). Subsequent ^18^F-FDG PET/CT resolved this challenge by identifying hypermetabolic foci (SUVmax 8.5) within the lesion, guiding a repeat biopsy to viable tumor regions that confirmed SMARCA4 loss (BRG1-negative, Ki67 70%). Such metabolic mapping is critical, as SMARCA4-deficient undifferentiated tumors often present with heterogeneously enhancing masses where CT alone cannot reliably distinguish between necrotic and metabolically active tumor compartments (7). Early and accurate diagnosis remains imperative, given the tumor’s rapid progression and dismal prognosis (median survival <12 months with conventional therapies) (8). This case underscores the limitations of anatomic imaging-guided biopsy in SMARCA4-deficient undifferentiated tumors and highlights the indispensable role of metabolic imaging in securing timely histologic diagnosis, thereby enabling prompt initiation of tailored multimodal therapy.

Beyond diagnostic hurdles, the aggressive biology of SMARCA4-deficient undifferentiated tumors necessitates innovative therapeutic strategies (9). Emerging evidence underscores the survival benefits of multimodal regimens over conventional chemotherapy alone. Mechanistically, this synergy may arise from chemotherapy-induced immunogenic cell death, enhanced T-cell infiltration via VEGF inhibition, and sustained immune activation through PD-L1 blockade (10). Such multimodal approaches are particularly relevant given the tumor’s rarity, resistance to conventional protocols, and propensity for early metastatic spread (11). This case illustrates that multimodal therapy combining chemotherapy, immunotherapy, anti-angiogenesis, and radiotherapy may offer transient benefit.

## Case report

### Clinical presentation and initial diagnosis

**Figure 1:**
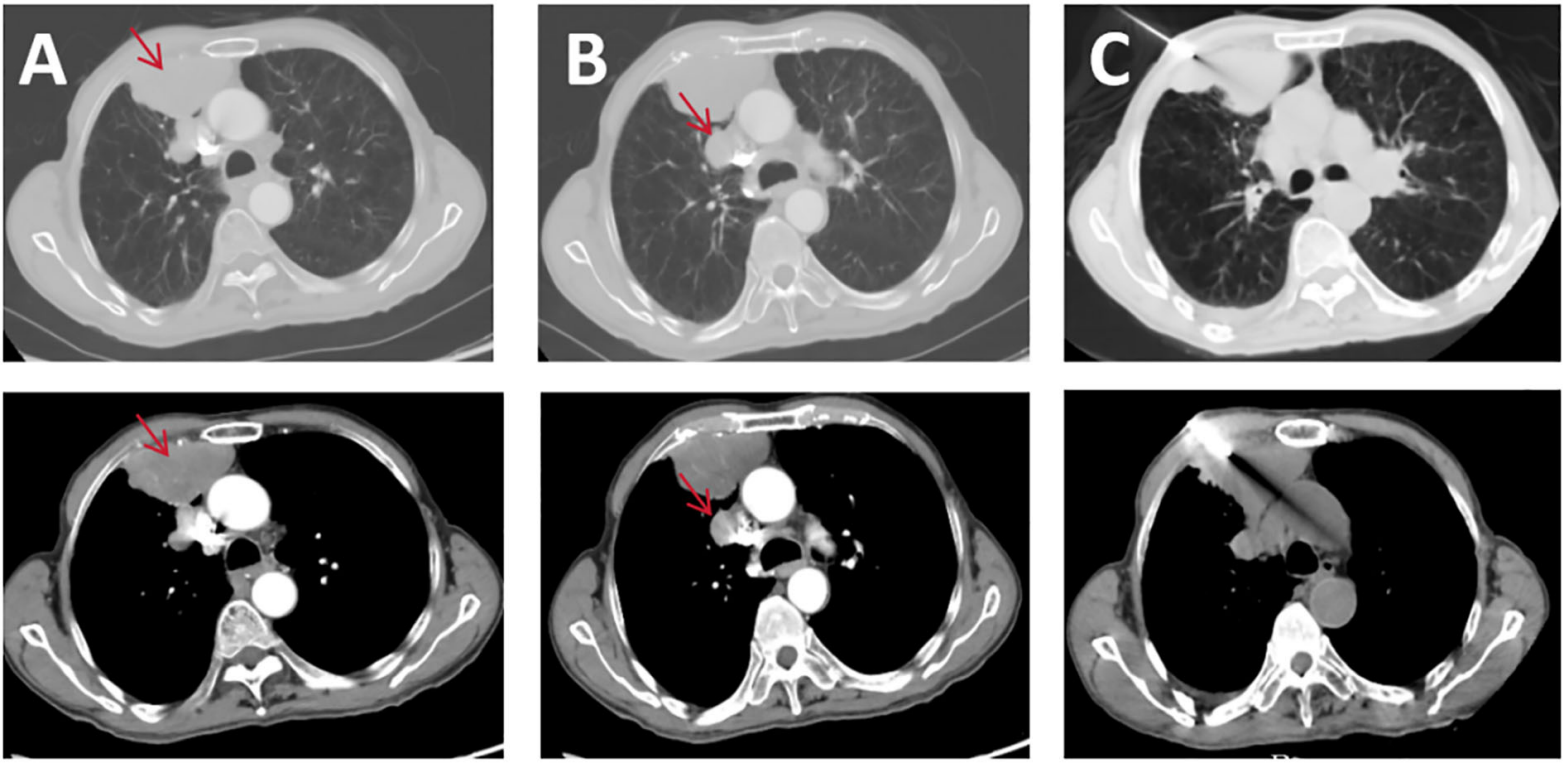
Pre-treatment CT scans: **(A)** pleural lesion lung window and mediastinal window; **(B)** mediastinal lymph node lesion lung window and mediastinal window; **(C)** puncture biopsy CT image.

A 74-year-old man with a 100-pack-year smoking history presented with right knee pain after a ground-level fall. Initial radiographs demonstrated a comminuted fracture of the right patella. Surveillance contrast-enhanced chest CT revealed a 6.8 × 3.9cm heterogeneously enhancing mass in the right anterior pleura (**Figure 1A**, lung/mediastinal window), accompanied by ipsilateral hilar lymphadenopathy (**Figure 1B**, lung/mediastinal window). A CT-guided core needle biopsy targeting the central region of the pleura mass (**Figure 1C**, puncture site) yielded necrotic debris without viable tumor cells, consistent with the high central necrosis rate (30–45%) in SMARCA4-deficient sarcomas.

**Figure 2:**
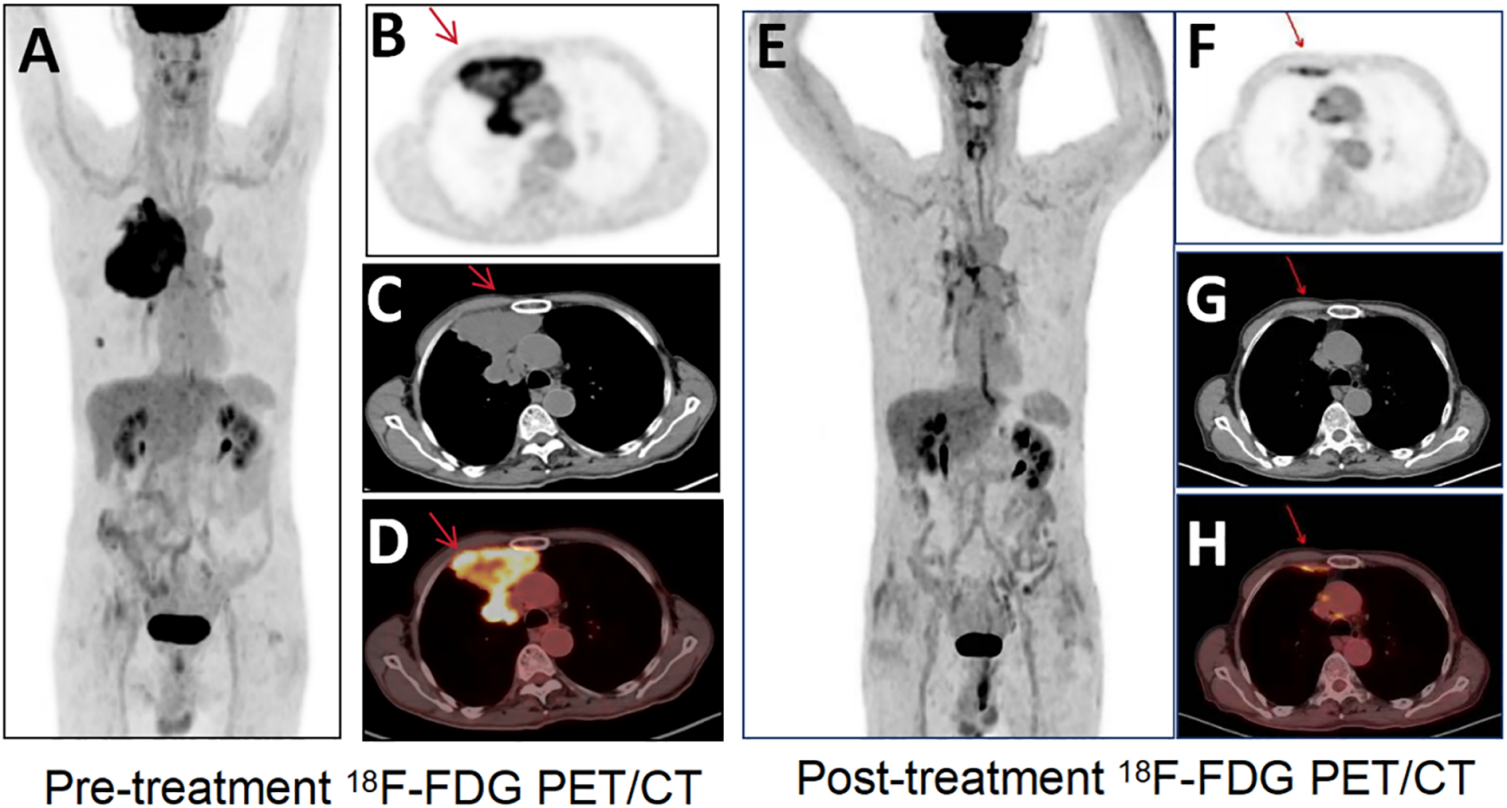
^18^F-FDG PET/CT images. **(A)** Pre-treatment ^18^F-FDG PET/CT shows high metabolic activity. ^18^F-FDG PET/CT transverse images [**(B)** PET; **(C)** CT; **(D)** fusion] show an irregular soft-tissue-density mass in the anterior-superior region of the right pleura, with a maximal cross-sectional area of approximately (SUVmax 8.2), and a right lung nodule, pleural deposits, and mediastinal lymph nodes. Transverse image **(E)** Post-treatment ^18^F-FDG PET/CT shows decreased metabolic activity of the primary tumor (SUVmax 2.7) and regression of the nodule/pleural metastasis. Transverse images [**(F)** PET; **(G)** CT; **(H)** fusion] of ^18^F-FDG PET/CT show the corresponding tumors (arrows).

**Figure 3:**
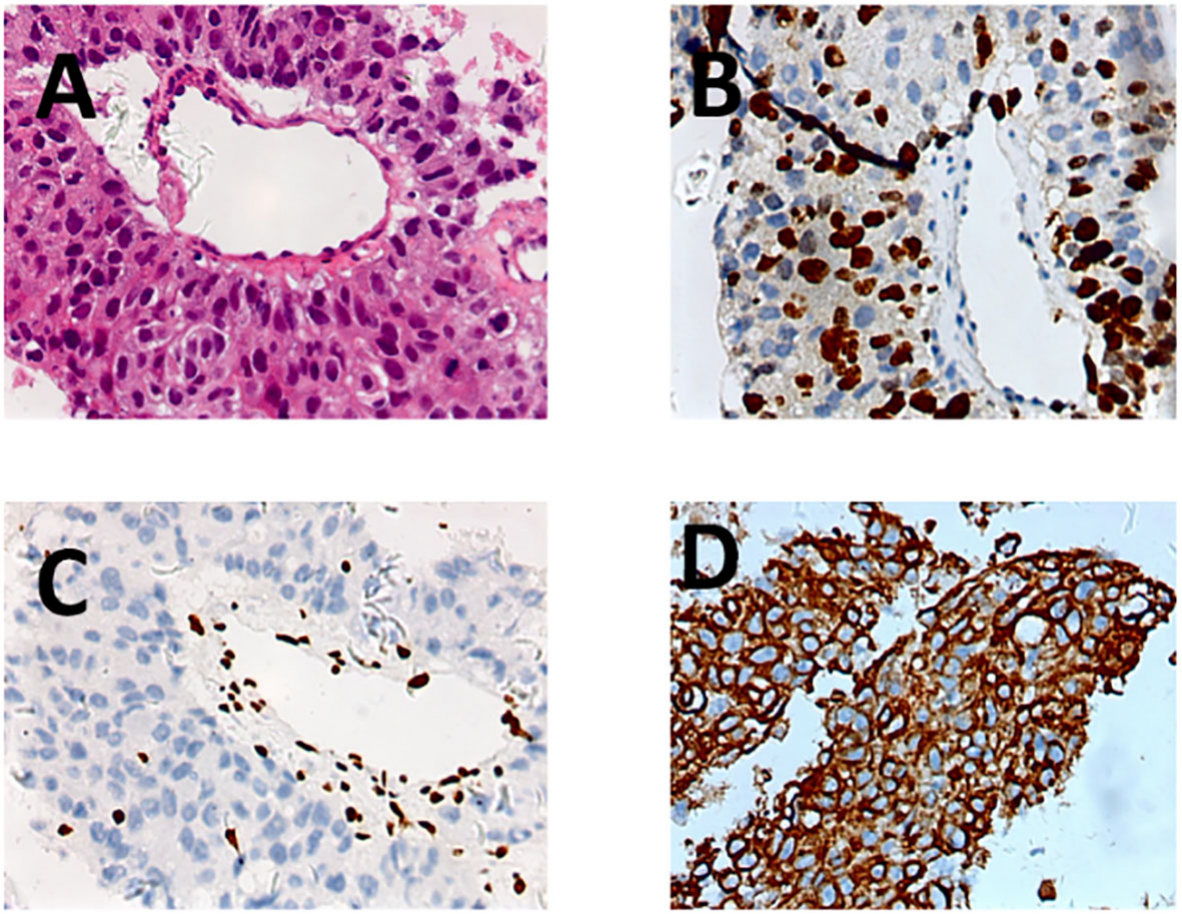
Puncture biopsy pathology immunohistochemistry. **(A)** SMARCA4-deficient undifferentiated tumor HE staining; **(B)** SMARCA4-deficient undifferentiated tumor positive Ki67 expression **(C)** SMARCA4-deficient undifferentiated tumor negative BRG1 expression **(D)** SMARCA4-deficient undifferentiated tumor PCK expression.

^18^F-FDG PET/CT resolved this diagnostic impasse by identifying intense peripheral hypermetabolism within the mass (SUVmax 8.2) (**Figure 2A**, pre-treatment PET) and metabolically active pulmonary nodules (SUVmax 6.3), pleural deposits (arrow), and mediastinal nodes (**Figures 2B-D**, fusion CT/PET). Guided by these metabolic hotspots, a repeat biopsy of the tumor periphery confirmed sheets of undifferentiated malignant cells with rhabdoid morphology (**Figure 3A**, H&E), showing loss of BRG1 immunohistochemical stain (**Figure 3C**), diffuse Ki67 index of 70% (**Figure 3B**), and pan-cytokeratin expression (PCK+, **Figure 3D**), establishing the diagnosis of SMARCA4-deficient undifferentiated tumor. Molecular profiling ruled out targetable EGFR/ALK/ROS1 alterations, and the PD-L1 combined positive score (CPS) was 15.

### Staging and treatment

PET/CT upstaged the disease to cT4N2M1a (IVA), with disseminated metastases to the lung, pleura, and mediastinal nodes (**Figures 2B-D**). Given the aggressive biology and inoperability, a multidisciplinary team initiated combined palliative therapy: nanoparticle albumin-bound paclitaxel (300 mg/m²) + carboplatin (AUC5) q3w, anlotinib (12 mg/day), and PD-L1 inhibitor bemtizumab (1200 mg q3w). Post four cycles, restaging PET/CT showed a 67% reduction in primary tumor metabolic activity (SUVmax 2.7, **Figures 2E-H**) and complete resolution of nodal/pleural metastases (arrowheads, **Figure 2E**). A partial response (RECIST v1.1) was achieved. Consolidative radiotherapy targeted the primary site (40 Gy/10 fx) and residual nodes (55 Gy/25 fx), maintaining disease control at an 11-month follow-up.

### Outcome

At an 11-month follow-up, the patient maintained CR with improved performance status (ECOG 1). Repeat CT imaging showed no new lesions. The combination of PET/CT-guided therapy and sequential chemoradiotherapy achieved durable local and systemic control (**Supplementary Figure S1**).

## Discussion

SMARCA4-deficient undifferentiated tumors account for <1% of thoracic malignancies and predominantly affect middle-aged to elderly men with a history of heavy smoking (10). Mutation in the SMARCA4 gene, a critical ATPase subunit of the SWI/SNF chromatin remodeling complex, leads to global epigenetic dysregulation, impaired differentiation, and aggressive tumor biology (2, 12). Histologically, these tumors mimic undifferentiated carcinoma or sarcomatoid mesothelioma, often delaying diagnosis due to non-specific imaging features and frequent biopsy sampling errors due to necrosis (13). In this case, the initial CT-guided biopsy failed to yield diagnostic material, a recurring challenge in SMARCA4-deficient tumors where >30% of cases require repeat biopsies to obtain viable tissue for immunohistochemical confirmation (14).

^18^F-FDG PET/CT is a valuable tool for initial diagnosis, staging, and treatment response assessment in aggressive thoracic malignancies, particularly in SMARCA4-deficient undifferentiated tumors where conventional imaging often fails to capture the full disease burden (15). In this case, PET/CT outperformed contrast-enhanced CT in two critical aspects. The initial contrast-enhanced CT localized the tumor to the right anterior pleura but was ambiguous in suggesting an overlapping pleural or pulmonary origin. ^18^F-FDG-PET/CT resolved this uncertainty by mapping hypermetabolic activity (SUVmax 8.2) to the pleural surface and adjacent nodal stations, a distinction critical for histological targeting and surgical planning. SMARCA4-deficient undifferentiated tumors often present as large necrotic masses with ill-defined margins on CT, mimicking pleural mesothelioma or undifferentiated carcinoma. PET metabolic profiling helps to prioritize biopsy sites to avoid sampling errors in necrotic zones, as seen in this patient’s initial non-diagnostic biopsy (16). While operator experience may influence yield, the high necrosis rate in SMARCA4-deficient undifferentiated tumors remains the primary contributor to sampling error. In addition, PET/CT revealed occult pleural deposits and subcentimeter nodal metastases (e.g., mediastinal nodes) missed by CT (**Figure 1A**), upstaging the disease to IVA and changing the therapeutic intent from curative to palliative. This is consistent with studies showing that 30-40% of SMARCA4-deficient cases have unsuspected distant metastases at diagnosis, necessitating PET/CT for accurate staging (17). Post-chemotherapy PET/CT showed a 67% reduction in SUVmax (8.2 → 2.7), classifying the response as partial metabolic remission (PERCIST criteria). This metabolic improvement preceded anatomical shrinkage, confirming the superior sensitivity of PET in detecting early treatment effects. Notably, residual metabolic activity in the pleural lesion guided radiotherapy targeting, ensuring dose escalation to viable tumor foci while sparing adjacent lung parenchyma. Such precision is critical in SMARCA4-deficient tumors, which exhibit heterogeneous radioresistance and a propensity for local recurrence. The integration of metabolic data with RECIST 1.1 criteria increased clinical confidence in maintaining immunotherapy despite transient risks of pseudoprogression, a phenomenon increasingly recognized in PD-L1 inhibitor-treated sarcomas (18, 19).

SMARCA4-deficient undifferentiated tumors have a poor prognosis; current guidelines lack standardized therapeutic algorithms for SMARCA4-deficient undifferentiated tumors, with median progression-free survival (PFS) with conventional chemotherapy reported to be 3–4 months (20, 21). This case highlights the potential of combining chemotherapy, immunotherapy, and radiotherapy to overcome therapeutic resistance through three synergistic mechanisms. Deficiency of the SWI/SNF complex induces three key vulnerabilities that can be exploited by multimodal approaches. SMARCA4 loss disrupts DNA repair via defective homologous recombination (HR) and mismatch repair (MMR), increasing tumor mutation burden (TMB) by 10–15 mutations/Mb compared to SMARCA4-intact sarcomas (22, 23). A Mutation in the SMARCA4 gene (BRG1) induces genomic instability and high tumor mutational burden (TMB), promoting neoantigen expression amenable to immune checkpoint inhibitors. Benmelstobart, a PD-L1 inhibitor, likely enhances T-cell infiltration by disrupting the PD-L1/CD80 “immune synapse hijacking” observed in SMARCA4-deficient tumors (24, 25). This promotes neoantigen expression, priming tumors for PD-L1 inhibitors such as bemotuzumab, which reverses T-cell depletion by disrupting PD-L1/CD80 co-inhibitory signalling (26). Dysregulated SWI/SNF activity upregulates hypoxia-inducible factor 1α (HIF-1α) and VEGF-A, creating tortuous, hyperpermeable vessels that impair drug delivery (27). Anlotinib, a multitarget tyrosine kinase inhibitor (TKI), selectively inhibits VEGFR2/3, FGFR1–4, and PDGFR-α/β. Preclinical studies reveal that SMARCA4-deficient tumors recruit abnormal neovasculature with heightened VEGF expression, contributing to hypoxic niches resistant to cytotoxic drugs (28, 29). By “normalizing” tumor vasculature, anlotinib improves tumor perfusion and gemcitabine/carboplatin penetration, as evidenced by the rapid metabolic response here (PR after 2 cycles). This aligns with phase II trials showing anlotinib-carboplatin combinations improve progression-free survival (PFS) in thoracic sarcomas by 3.2 months compared to chemotherapy alone (30). Preclinical models demonstrate that SMARCA4-deficient cells exhibit 2.5-fold increased radiation sensitivity due to defective HR repair and ATM/ATR signaling. Consolidative radiation synergizes with PD-L1 blockade by activating the cGAS-STING pathway, amplifying systemic antitumor immunity (31). Consolidative radiotherapy likely augmented systemic immunity through three mechanisms: Radiation-induced tumor antigen release and dendritic cell activation may have amplified Benmelstobart’s efficacy, potentially controlling micrometastases beyond irradiated fields (32). SMARCA4 loss impairs DNA repair, increasing cytosolic DNA accumulation post-radiation. This stimulates the STING pathway, enhancing interleukins (e.g., IFN-γ) and T-cell priming. Radiation-induced reoxygenation mitigates hypoxia-driven PD-L1 upregulation, restoring T-cell cytotoxicity (33, 34).

## Conclusion

**Figure 4:**
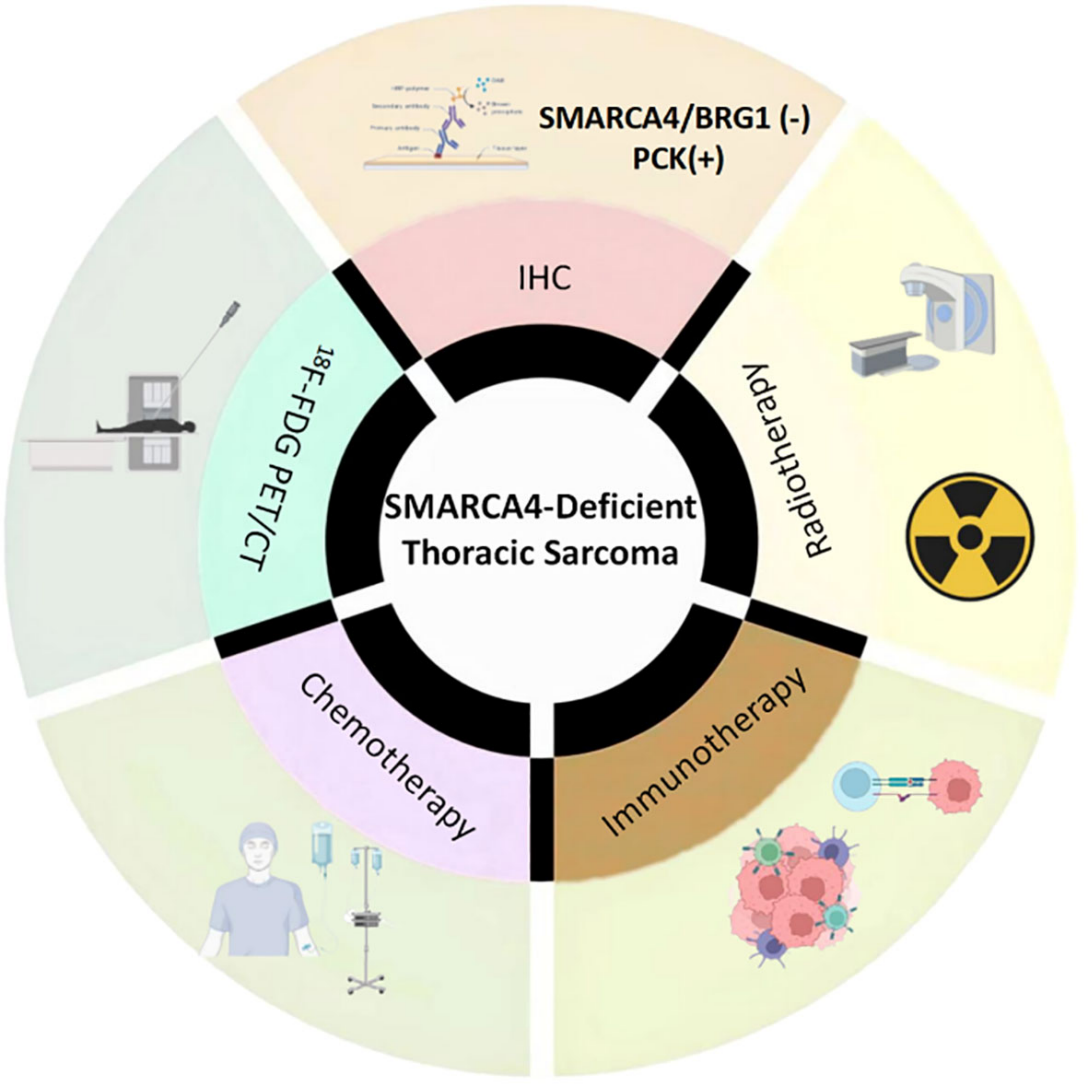
SMARCA4-deficient undifferentiated tumor: diagnostic and therapeutic workflow: ^18^F-FDG PET/CT metabolic mapping and puncture instruction, integration of IHC diagnosis, and combined chemotherapy, immunotherapy, radiotherapy in managing SMARCA4-deficient undifferentiated tumor.

SMARCA4-deficient undifferentiated tumor demands prompt diagnosis and aggressive treatment. ^18^F-FDG PET/CT is invaluable for staging and response assessment, ensuring precision in multidisciplinary care. The combination of chemotherapy, anti-angiogenics, immunotherapy, and radiotherapy may prolong survival in this otherwise dismal malignancy(**Figure 4**). Further studies are warranted to validate this approach.

This case illustrates the diagnostic complexity and aggressive nature of SMARCA4-deficient undifferentiated tumors. Multimodal therapy, including antiangiogenic agents, may offer transient benefit. Rapid molecular testing and multidisciplinary approaches are essential for optimizing outcomes.

## Data Availability

The original contributions presented in the study are included in the article/**Supplementary Material**. Further inquiries can be directed to the corresponding authors.
