# Supplementary material for: Case Report: Synergistic multimodal therapy in SMARCA4-deficient undifferentiated tumor: integrating chemotherapy, anti-angiogenesis immunotherapy, and radiotherapy for enhanced outcomes
Source: Front Oncol. 2025 Sep 5;15:1599569. doi: 10.3389/fonc.2025.1599569 (PMC12446001; doi:10.3389/fonc.2025.1599569)
Supplement: Supplementary file 1 [file Image1.pdf]

Supplementary materials

Synergistic Multimodal Therapy in SMARCA4-deficient undifferentiated tumor: Integrating Chemotherapy, Anti-Angiogenesis Immunotherapy, and Radiotherapy for Enhanced Outcomes

Fang Xie[1], Yuming Jia[1], Kaijian Lei[1], Zhongming Wang[4], Wei Zhang[5], Shiyu Zheng[3, *], and Daohong Kan[2, *]

[1] Department of Oncology, The Second People's Hospital of Yibin, Yibin, Sichuan, China,

[2] Department of Burn and Plastic Surgery, The Second People's Hospital of Yibin, Yibin, Sichuan, China,

[3] Department of Nuclear Medicine, The Second People's Hospital of Yibin, Yibin, Sichuan, China,

[4] Department of Internal Medicine, Gongxian Hospital of Traditional Chinese Medicine, Yibin, Sichuan, China

[5] Department of Rehabilitation, Gongxian Hospital of Traditional Chinese Medicine, Yibin, Sichuan, China

*** Corresponding authors:**

Daohong Kan

kdh4639@163.com

Shiyu Zheng

215879192@qq.com

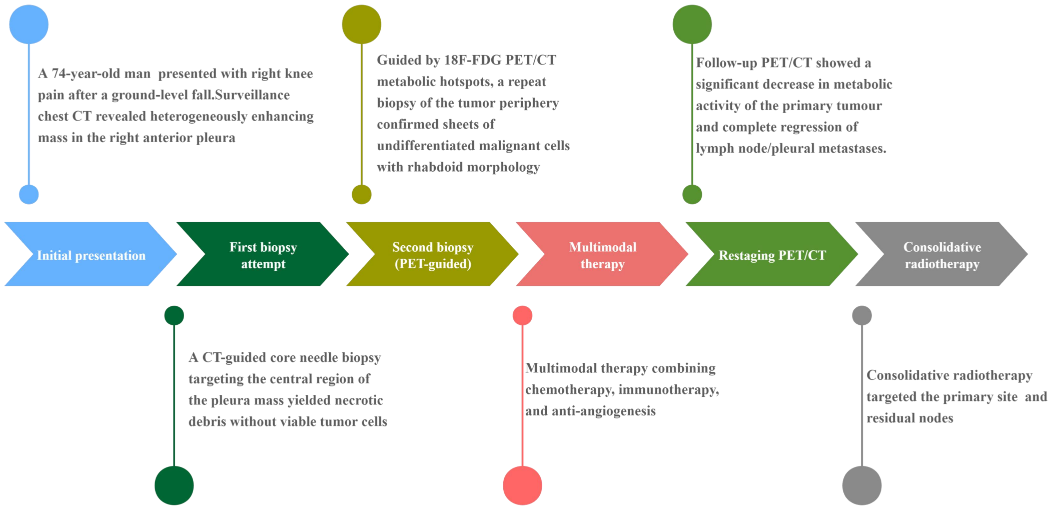

Figure S1 Chronological clinical timeline of SMARCA4-Deficient undifferentiated tumor management
